# Benign Paroxysmal Positional Vertigo and Occult Subarachnoid Hemorrhage Complicated after Head Trauma

**DOI:** 10.1155/2020/8507383

**Published:** 2020-03-30

**Authors:** Qinghua Li, Shuangxing Hou, Hualan Yang

**Affiliations:** Department of Neurology, Shanghai Pudong Hospital, Fudan University Pudong Medical Center, 2800 Gongwei Road, Shanghai, China

## Abstract

Benign paroxysmal positional vertigo (BPPV) is the most prevalent form of peripheral vertigo and is common in posttraumatic patients. Sometimes, posttraumatic BPPV and subarachnoid hemorrhage (SAH) exist together. How to effectively recognize SAH especially concealed bleeding before maneuver treatment for BPPV is worth paying attention by every clinician. Presently described is a case that when there are some clinical symptoms cannot be completely explained by simple BPPV, the combination of CT and FLAIR MRI sequences are needed in the early-stage detection of acute SAH.

## 1. Introduction

Benign paroxysmal positional vertigo (BPPV) is the prevalent form of peripheral vertigo after head trauma. Maneuver is safe and effective in general patients for diagnosis and treatment. However, high systolic blood pressure induced by dizziness, nausea, and vomiting in the process of maneuvers will increase the risk of rebleeding in intracerebral hemorrhage patients, even a case reported a patient experienced a hemorrhagic stroke after undergoing the Epley maneuver [1, 2]. With a reliable medical history given by patient, we need to differentiate central diseases, to avoid missing other dangerous diagnosis and to give a maneuver safely. Usually, CT can effectively help us identify bleeding, but sometimes there is a false negative. Presently described is a case that tells us that sometimes the combination of MRI FLAIR sequence is better in detecting early-stage acute occult subarachnoid hemorrhage.

## 2. Case Report

A 69-year-old male patient with history of a fall from a balcony presented at the neurology clinic with complaints of dizziness, tired, and slow walking persisting for 1 day. He had no memory of the way of fall. He only remembered when he woke up; he was lying on the ground with pain on the head, neck, and right leg. Then, he went back home by bike. He felt so tired that he went to sleep until the next morning.

**Figure 1:**
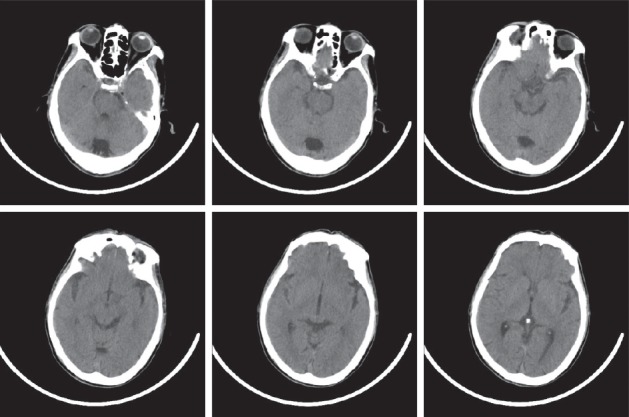
The CT image in July 28th. No blood could be seen.

After a good rest at night, he felt less painful (only a little neck soreness), but paroxysmal dizziness, which was provoked by sudden movements, especially when lying down and getting up, and is often accompanied by nausea. In addition, no positive signs were found during the neurological physical examination. But there is a sense of slight pain when doing Kernig or Brudzinski test. A head CT scan (Figure 1) and electroencephalogram were done firstly because there is a hematoma on the scale, making us hypothesizing the patient had a head trauma. Except for soft tissue hematoma, the results of CT and electroencephalogram were normal. Then, Dix-Hallpike test was done and Epley's maneuver was performed for right posterior canal BPPV.

**Figure 2:**
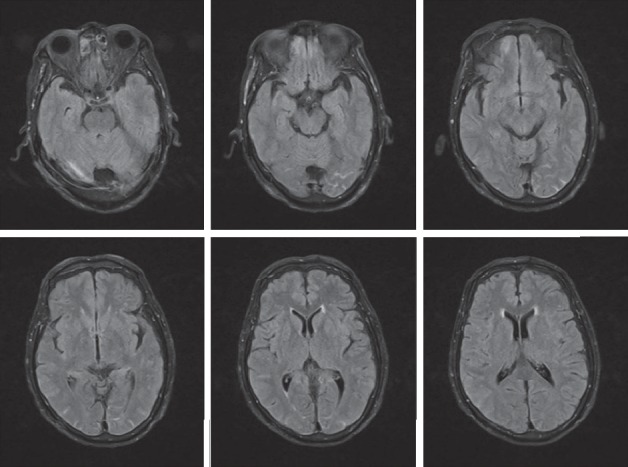
The MRI image in July 31st. A small amount of bleeding was displayed in the occipital lobe.

**Figure 3:**
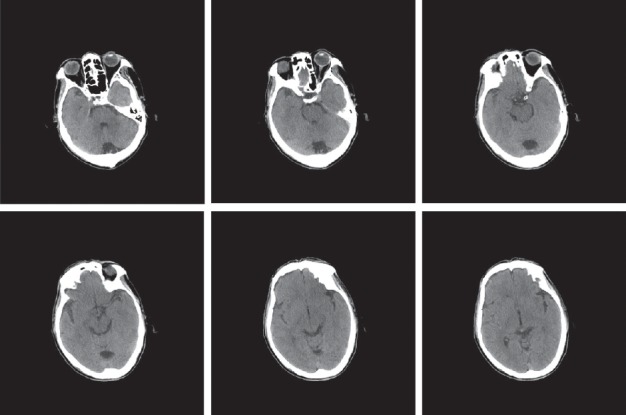
The CT examination in August 2nd. No blood could be seen.

Two days later, he still had an uncomfortable felling including punch-drunk with fatigue, drowsiness, slow walking, and neck soreness. Then, a head magnetic resonance imaging (MRI) was done on 31st July, and the Flair phase showed a high density in the partial sulci of the occipital lobe (Figure 2). Then, CT was done again on 2nd August (Figure 3), and still no blood was seen. And a lumbar puncture was made quickly and three tubes of homogeneous and bloody cerebrospinal fluid were got, which helped us make a definite diagnosis of subarachnoid hemorrhage. Finally, digital subtraction angiography (DSA) was performed. However, no aneurysm or arteriovenous malformations were detected.

## 3. Discussion

SAH is an important type of hemorrhagic cerebrovascular disease, and early recognition and appropriate treatment should be paid great attention because misdiagnosis may lead to serious consequences. Usually, typical bleeding has obvious clinical symptoms such as severe headache, nausea, and vomiting, and CT examination can easily give a diagnosis of bleeding. However, the occult SAH such as little bleeding located in the posterior cranial fossa is easy to be missed. Especially when BPPV and SAH co-occur after a head trauma, the risk of rebleeding is increased by high systolic blood pressure induced by maneuver [1, 2].

Head CT is commonly performed acutely for the evaluation of traumatic SAH, because its sensitivity is 90–95% in 24 hours, 80% in 3 days, and 50% in 1 week [3, 4]. But a little bleeding is easy to be missed by CT when it is located in the posterior cranial fossa. However, MRI has an excellent sensitivity for the detection of acute intracranial hemorrhage in early stage, and traumatic SAH may be identified as hyperintense signal abnormality overlying the cerebral sulci on fluid-attenuated inversion recovery (FLAIR) sequences or hypointense susceptibility blooming on Gradient-Echo (GRE) or susceptibility-weighted imaging (SWI) sequences. And MR may play a greater role when the sensitivity of CT decreases several days after the disease. After 4 days, T1 images clearly showed extravasation of blood, with high blood signals lasting for at least 2 weeks, while FLAIR images lasted longer [4–6]. After we found that someone has persistent neck soreness, drowsiness, and slow walking and cannot be completely explained by BPPV, the combination of CT and early FLAIR MRI sequences are needed in the detection of early-stage SAH.

Although most subarachnoid hemorrhages are traumatic in nature, DSA should be done in SAH patients to remove the possibility of intracranial arteriovenous malformation (AVM) or aneurysm because many patients surviving an ASAH/AVM will have long-term functional and cognitive impairments [7–9], which is the gold standard for the diagnosis of aneurysms or AVM.
